# Visual exposure to large and small portion sizes and perceptions of portion size normality: Three experimental studies

**DOI:** 10.1016/j.appet.2015.12.010

**Published:** 2016-03-01

**Authors:** Eric Robinson, Melissa Oldham, Imogen Cuckson, Jeffrey M. Brunstrom, Peter J. Rogers, Charlotte A. Hardman

**Affiliations:** a Department of Psychological Sciences, Eleanor Rathbone Building, University of Liverpool, Liverpool, UK; b Nutrition and Behaviour Unit, School of Experimental Psychology, University of Bristol, UK

**Keywords:** Portion size, Visual adaptation, Norms, Visual exposure, Food selection

## Abstract

Portion sizes of many foods have increased in recent times. In three studies we examined the effect that repeated visual exposure to larger versus smaller food portion sizes has on perceptions of what constitutes a normal-sized food portion and measures of portion size selection. In studies 1 and 2 participants were visually exposed to images of large or small portions of spaghetti bolognese, before making evaluations about an image of an intermediate sized portion of the same food. In study 3 participants were exposed to images of large or small portions of a snack food before selecting a portion size of snack food to consume. Across the three studies, visual exposure to larger as opposed to smaller portion sizes resulted in participants considering a normal portion of food to be larger than a reference intermediate sized portion. In studies 1 and 2 visual exposure to larger portion sizes also increased the size of self-reported ideal meal size. In study 3 visual exposure to larger portion sizes of a snack food did not affect how much of that food participants subsequently served themselves and ate. Visual exposure to larger portion sizes may adjust visual perceptions of what constitutes a ‘normal’ sized portion. However, we did not find evidence that visual exposure to larger portions altered snack food intake.

## Highlights

• Three studies examined visual exposure to large portion sizes.
• Exposure to larger portions altered perceived normality of portion size.
• Exposure to larger portions altered ideal meal size.
• Exposure to larger portions did not alter immediate food consumption.

## 1 Introduction

Food portion sizes have increased for a number of food types in recent years (Nielsen and Popkin, 2003, Smiciklas-Wright et al., 2003). This could be problematic because larger portion sizes are associated with increased energy intake (Benton, 2015, Jeffery et al., 2007). Work by Vartanian and colleagues suggests that portion size may influence food consumption because it signals a type of social norm about what is an appropriate amount to consume (Herman et al., 2015, Kerameas et al., 2014). Recent work by Marchiori et al. also suggests that portion size may act as a cue or ‘norm’ which influences meal size (Marchiori, Papies, & Klein, 2014), such that when making evaluations about portion size, individuals anchor their decisions relative to the size of the portion size being evaluated. In support of this ‘norm’ based or ‘anchoring’ account, studies have shown that portion sizes can differ significantly in size whilst still being rated as equally ‘normal’ or appropriate (Diliberti et al., 2004, Robinson et al., 2015).

Although we know that portion sizes of some foods have increased (Nielsen and Popkin, 2003, Smiciklas-Wright et al., 2003), little research has examined the psychological consequences of being exposed to larger portion sizes. A body of research now suggests that perceived normality of stimuli can be influenced by visual learning, otherwise known as a visual adaptation effect. There is evidence that frequent visual exposure to large variants of a stimulus type can result in a recalibration of what range of that stimulus is perceived as being ‘normal’ in size (Boothroyd et al., 2012, Winkler and Rhodes, 2005), as well as a person's preferred body size (Robinson and Christiansen, 2014, Winkler and Rhodes, 2005). For example, visual exposure to obese body shapes has been shown to alter perceptions of what a normal sized body looks like, whereby normal appears larger (Oldham and Robinson, 2015, Robinson and Kirkham, 2014). Thus, based on the visual adaptation literature, one possible consequence of increases in food portion sizes is that more frequent visual exposure to larger portion sizes recalibrates visual perceptions of what a ‘normal’ sized portion of food looks like. In line with this notion are a number of studies which have examined ‘portion distortion’ (Almiron-Roig et al., 2013, Schwartz and Byrd-Bredbenner, 2006). Portion distortion refers to the observation that consumers have a poor understanding of what constitutes a normal or appropriate sized food serving and the direction of this distortion is often indicative of overestimation (Almiron-Roig et al., 2013, Schwartz and Byrd-Bredbenner, 2006); namely that consumers overestimate what they think of as being a normal serving of food. Importantly, social eating research consistently indicates that information and perceptions about what constitutes a normal amount of food to eat can influence how much a person eats (Robinson et al., 2013, Robinson et al., 2014, Vartanian et al., 2013). Therefore, a further consequence of exposure to larger portion sizes is that it may affect food intake by altering perceptions of what constitutes a normal sized portion of food.

The aim of the present research was to experimentally test the effect that visual exposure to larger versus smaller food portion sizes has on perceptions of what constitutes a normal portion size (studies 1–3), self-reported ideal portion size (studies 1–2) and food consumption (study 3). We hypothesised that visual exposure to large portion sizes would alter perceptions of what constitutes a normal sized portion (to be larger) and that this may also cause participants to select larger meal sizes, as individuals can be motivated to eat in line with what they believe to be a ‘normal’ amount to eat (Robinson et al., 2013, Robinson et al., 2014, Vartanian et al., 2013).

## 2 Study 1

In study 1 participants were visually exposed, via an internet-delivered questionnaire, to ten images of large or small portion sizes of spaghetti bolognese or non-food objects (control). After this initial exposure phase participants were shown an intermediate portion size of spaghetti bolognese and indicated whether they believed a ‘normal’ serving of spaghetti bolognese was smaller or larger than the intermediate portion size presented. They then reported what their ideal portion size of spaghetti would be (relative to the intermediate portion size presented). Participants also made the same evaluations about an intermediate portion size of a different food (chicken curry and rice), to allow us to examine whether any visual exposure effects may transfer to a different (non-congruent) food type.

### 2.1 Participants

One hundred and fifty (113 female, 37 male) university students and staff (M age = 39.0 yrs, SD = 11.6) completed an online study about ‘Personality and Perception’ and were entered into a small prize draw as reimbursement (M BMI calculated from self-report weight/height^2^ = 25.0, SD = 4.9 kg/m^2^). The study was approved by the University of Liverpool Research Ethics Committee (as was study 3).

### 2.2 Design and portion sizes

**Fig. 1:**
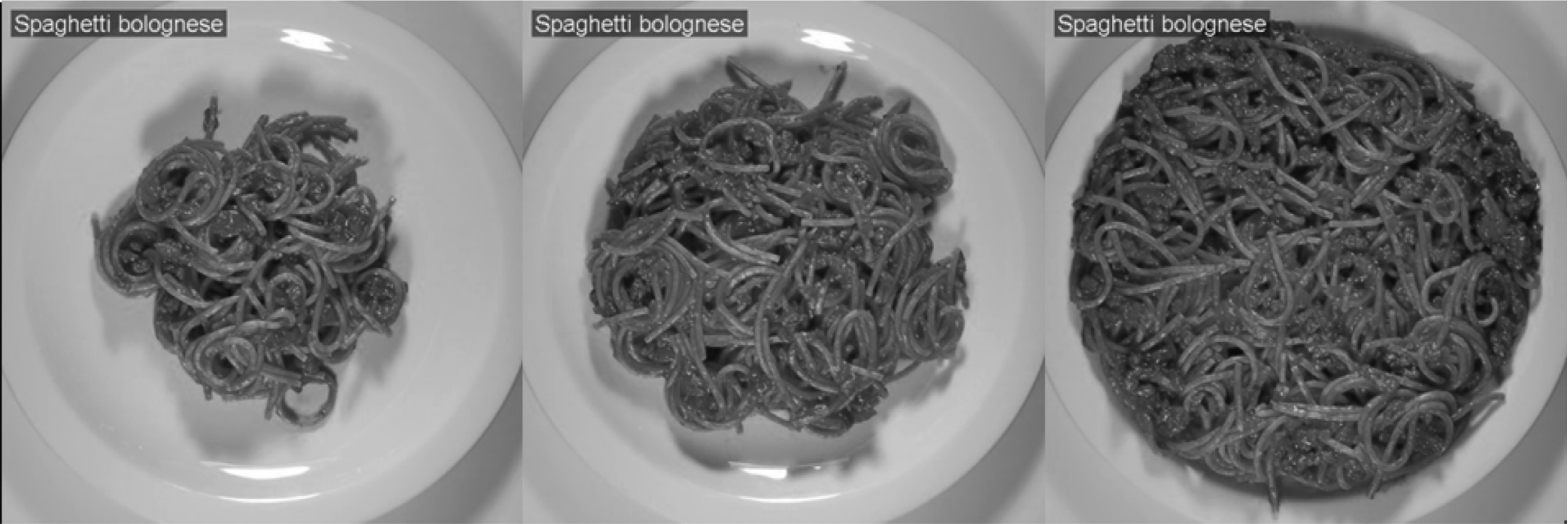
Example small portion size, intermediate portion size and large portion size images from study 1. See text for kcal content of portions presented in each condition.

A between-subjects design was used, with participants randomized into one of the three conditions. In the portion size exposure conditions participants were exposed to ten standardised photographs of the same plate containing either small or large servings of spaghetti bolognese. In the small portion size exposure condition the servings were between 340 and 420 kcal (M = 380 kcal) and in the large portion size exposure condition the servings were between 920 and 1000 kcals (M = 960 kcal). See Fig. 1 for example images. In the control condition participants were exposed to photographs of everyday objects (e.g. a sofa). We included this control condition for comparative purposes in order to detect the direction of any observed effect; e.g. it is feasible that visual exposure to small, but not large portion sizes could alter perceptions of the size of a normal portion of food. The intermediate portion size of spaghetti bolognese that all participants later evaluated was 520 kcal, as this portion size was approximately half way (in terms of food volume by the eye, as agreed upon by the research team) between the portion sizes in the small and large exposure conditions, as shown in Fig. 1.

### 2.3 Procedure

After logging onto the online study site and providing informed consent, participants were instructed that they would be rating a series of images and completing self-report measures. Participants were then randomized to one of the three conditions and rated ten images on consecutive pages. In the portion size conditions participants evaluated each image on dimensions unrelated to portion size (e.g. ‘how exotic does this look’) using a 0 (not at all) to 10 (extremely) visual analogue scale (VAS). In the control condition participants made similar ratings about everyday objects. After participants made each rating they continued onto the next image using a cursor on screen. Thus, the duration of exposure to each image was not pre-defined. In order to examine the effect of exposure to everyday objects (control) and small or large portions of spaghetti bolognese, the 11th and 12th images for all three conditions were always of the intermediate portion size of spaghetti bolognese. To measure *ideal portion size*, participants rated the 11th image using the same VAS: ‘If I were to eat this for an evening meal, I would want a portion size that was’, anchors: a lot smaller and a lot bigger. On the next page (12th image), to measure *perceived normality of portion size,* participants used the same scale to make the following rating: ‘A normal serving of spaghetti bolognese would be’, anchors: a lot smaller and a lot bigger. The 13th and 14th images presented were of an intermediate serving of the different food: chicken curry and rice (420 kcal). Participants made the same ratings as for images 11 and 12.

Participants next reported their age, gender, weight and height, as well as being asked ‘think back to just before you were about to start the study, how hungry were you? Options: not at all hungry, a little hungry, moderately hungry, and extremely hungry. These measure were included to examine whether the conditions were balanced for these variables. Finally, participants completed a shortened five-item version of the Restraint Scale of the Three Factor Eating Questionnaire (Stunkard & Messick, 1985), e.g. ‘ I count calories as a conscious means of controlling my weight’ which we included to check that conditions were balanced for dietary restraint (the 5 items were selected by the research team). At the end of the study participants were asked to guess the aims of the study, were offered the opportunity to be entered into the prize draw and were debriefed.

### 2.4 Analysis

One way ANOVA was used to check that conditions were balanced for baseline variables (Chi Square for gender) and to examine whether the exposure condition that participants were assigned to impacted on their evaluations of the intermediate portion sizes of spaghetti bolognese (congruent food) and chicken curry and rice (incongruent food). If an effect was observed in the ANOVA, planned pairwise comparisons were used to examine between condition differences.

### 2.5 Results

No participants directly guessed the aims of the study, although four participants reported that they believed the study may have been related to portion size normality (e.g. to examine ‘how much people think is a normal amount to eat in a meal?’). Removal of these participants did not affect any of the significant and non-significant results reported. There were no significant differences between conditions for age, BMI, gender, dietary restraint or hunger (all *ps* > .05).

#### 2.5.1 Food portion size evaluations (congruent)

ANOVA indicated a significant effect of condition on *perceived normality of portion size for* spaghetti bolognese [F(2, 147) = 15.4, p < .001, *np*^*2*^ = .17]. See Table 1. Participants exposed to the large portions of spaghetti bolognese believed a normal serving of spaghetti bolognese would be larger (relative to the intermediate sized portion) than did participants exposed to small portions of spaghetti bolognese [t (97) = 5.2, p < .001, *d* = 1.05] and participants in the control condition [t (96) = 2.0, p = .046, *d* = 0.41]. Additionally, participants in the small portion size exposure condition believed a normal serving of spaghetti bolognese would be smaller in size compared with the control condition [t (101) = 3.8, p < .001, *d* = 0.74]. A parallel pattern of results was observed for *ideal portion size* of spaghetti bolognese [F(2, 147) = 14.7, p < .001, *np*^*2*^ = .17]. Participants in the large portion size exposure condition rated their ideal portion size of spaghetti bolognese as being larger than participants in the small portion size exposure condition [t (97) = 5.4, p = <.001, *d* = 1.09] and the control condition [t (96) = 2.4, p < .002, *d* = 0.50]. Participants' ideal portion size of spaghetti bolognese was smaller in the small portion size exposure condition than in the control condition [t (101) = 3.0, p = .004, *d* = 0.59].

#### 2.5.2 Food portion size evaluations (incongruent)

There were no significant effects of condition on *perceived normality of portion size* [F(2, 147) = 1.3, p = .29, *np*^*2*^ = 0.02] or on *ideal portion size* [F(2, 147) = 0.4, p = .70, *np*^*2*^ = 0.005] for evaluations made about the image of chicken curry and rice. See Table 1 for means and standard deviations.

### 2.6 Conclusions

In line with our hypotheses, after visual exposure to larger portion sizes of spaghetti bolognese, participants believed that a normal sized portion was larger and reported a larger ideal portion size of spaghetti bolognese, than in comparison to the control condition. Likewise, when participants were exposed to small portion sizes, relative to the control condition, perceptions of what constitutes a normal and ideal portion size were also altered, resulting in participants reporting smaller ideal and smaller normal portion sizes. Thus, these findings suggest that visual exposure to different portion sizes may adjust visual perceptions of what constitutes a normal and ideal portion size. We found no evidence that this visual exposure effect transferred to a different food item (chicken curry and rice). However, a limitation of study 1 was that we did not counterbalance the order of food type images (spaghetti bolognese followed by chicken curry and rice). Thus, in study 2 we aimed to replicate the main findings of study 1 whilst counterbalancing the order of portion size evaluations and food type images.

## 3 Study 2

We used the same method as in study 1, although as our main interest was in how visual exposure to different sized portions influences evaluations we dropped the no portion size exposure (control) condition. We also designed the online study to fully counterbalance the order in which participants evaluated portion size normality and ideal portion size, as well as the order in which intermediate portion sizes of spaghetti bolognese and chicken curry and rice were presented after the initial exposure phase trials.

### 3.1 Participants and analysis

Fifty five participants (M age = 29.8 yrs, SD = 12.0) were recruited over email from staff, students and the local community of the University of Bristol (UK). The study was approved by the University of Bristol Research Ethics Committee. The sample's M BMI (calculated from self-report weight/height^2^) was 24.3 (SD = 7.3). Thirty nine participants were female and sixteen were male. Our main planned analysis involved comparing the two portion size exposure conditions on portion size evaluation scores (perceived normality of portion size and ideal portion size for the congruent and incongruent foods) using independent samples t-tests.

### 3.2 Results

No participants directly guessed the aims of the study. Two participants reported that they believed the study may have been related to portion normality. Removal of these participants did not affect any of the significant and non-significant results reported. There were no significant differences between the small (N = 27) and large (N = 28) portion size exposure conditions for age, BMI, gender, dietary restraint or hunger (all *ps* > .05).

#### 3.2.1 Food portion size evaluations (congruent)

Participants exposed to large portion sizes of spaghetti bolognese believed a normal serving of spaghetti bolognese would be larger than did participants exposed to small portions of spaghetti bolognese [t (53) = 3.0, p = .004, *d* = 0.78]. See Table 2. Participants in the large portion size exposure condition also rated their ideal portion size of spaghetti bolognese as being larger than participants in the small portion size exposure condition [t (53) = 3.9, p < .001, *d* = 1.07]. See Table 2.

#### 3.2.2 Food portion size evaluations (incongruent)

Participants exposed to large portions of spaghetti bolognese believed a normal serving of chicken curry and rice would be larger than participants exposed to small portions of spaghetti bolognese [t (53) = 2.6, p = .012, *d* = 0.74]. See Table 2. Participants in the large portion size exposure condition and the small portion size exposure conditions did not significantly differ in their ratings about ideal portion size of chicken curry and rice [t (53) = 1.7, p = .10, *d* = 0.42]. See Table 2.

### 3.3 Conclusions

In line with the findings of study 1, after visual exposure to larger portion sizes of spaghetti bolognese, in comparison to visual exposure to smaller portion sizes, participants believed that a normal sized portion was larger and reported a larger ideal portion size of spaghetti bolognese. Unlike study 1 we did find some evidence of a transfer effect; exposure to larger portion sizes of spaghetti bolognese were associated with an increase in the size of the portion of chicken curry and rice participants reported as being ‘normal’. One interpretation is that exposure to a large portion of food may normalise larger portion sizes (i.e. fuller plates) more generally. However, it is important to note that we did not observe a significant transfer effect of exposure on ideal portion size in study 2, nor did we observe any significant transfer effects in study 1. Thus, it may be the case that any transfer effect of exposure to larger portion sizes on evaluations made about an incongruent food is weaker and less reliable than when a congruent food is being evaluated.

## 4 Study 3

The aim of study 3 was to replicate the effect that visual exposure to larger portions sizes has on portion size normality for a different food type (crisps) and to also examine whether exposure to larger portions of a food influences portion size selection and consumption in the laboratory (as opposed to self-reported ideal portion size). We recruited young adult females only in study 3 in order to reduce variability in food consumption across participants due to gender differences in food intake.

### 4.1 Participants

68 female participants (M age = 20.0 yrs, SD = 2.8) were recruited into a study about snacking and attitudes to snack food in exchange for course credit (psychology students) or a small monetary incentive. M BMI = 22.3 kg/m^2^ (SD = 3.9).

### 4.2 Design and portion sizes

A between-subjects design was used, with participants randomized to a large or a small portion size exposure condition. Participants were exposed to ten standardised photographs of the same plate containing either small or large servings of crisps. The photographs were of different brands of crisps, as we reasoned that participants may have become suspicious of the true purpose of the study if only the same type of crisp was rated on each page of the questionnaire. In the small portion size exposure condition the servings of crisps covered a small amount of the middle of the plate and were between 34.1 and 82 kcal (M = 62 kcal). In the large portion size exposure condition the servings covered the majority of the plate and were between 223 and 359 kcals (M = 295 kcal). The intermediate portion size of crisps that all participants later evaluated was of a serving of ready salted crisps (a large or small portion of this same brand of crisps was displayed in the initial exposure phase also). The portion size displayed (124 kcal) was approximately half way (by eye) between the portion sizes shown in the small and large exposure conditions.

### 4.3 Procedure

Sessions took place during weekday mornings (10am to midday) and participants were informed that the study would involve consuming a snack food during the session. Participants were randomized into one of the two portion size exposure conditions on arrival. After providing informed consent and confirming that they did not have any food allergies, participants completed a short questionnaire in which they recorded their age and gender, and the time they last ate. Next participants completed a set of mood ratings (e.g., ‘I am tired’, 0–10 cm VAS, anchors: not at all, extremely) and embedded into these items was a measure of baseline hunger (‘I am hungry’). Participants were then informed that the study would involve making ratings about different types of crisps and that they would later be asked to consume and rate a serving of crisps. Participants were then provided with the portion size exposure booklet, which consisted of the ten photographs of crisps described earlier (large or small in portion size). On a separate sheet participants made a rating on a 0–10 VAS scale about each image (e.g. how flavour some does this food look? anchors: not at all and extremely), as in studies 1 and 2. The 11th photograph was of the intermediate portion size and participants rated ‘a normal serving of crisps would be?’ 0–10 cm VAS, anchors: a lot smaller than this and a lot bigger than this. After completing the questionnaire, participants were provided with a glass of water and a large bowl of ready salted crisps (150 g, 526 kcal/100 g). Participants were asked to serve themselves a ‘serving’ of crisps to eat as a snack and were provided with a questionnaire in order to evaluate the crisps. To corroborate the cover story the questionnaire included items on the sensory dimensions of the crisps (e.g. how crunchy were the crisps? 0–10 cm VAS). After participants had selected a serving the researcher removed the serving bowl. After consuming the crisps and completing the evaluation questionnaire, participants were provided with a second set of mood ratings and the full Dietary Restraint and Disinhibition scales of the Three Factor Eating Questionnaire (Stunkard & Messick, 1985). In addition, participants completed a questionnaire which included an item on how frequently participants consumed crisps (6 point scale ranging from ‘every day’ to ‘monthly’) as well as an item which asked participants to guess the aims of the study. Participants then had their weight and height measured before being debriefed.

### 4.4 Analysis

We compared the two portion size exposure conditions for perceived normality of portion size and grams of crisps consumed using independent samples t-tests. The amount of food participants served and consumed was very similar (r = 0.81), so we report amount consumed only.

### 4.5 Results

No participants directly guessed the aims of the study, although seven participants reported that they believed the study may have been related to portion size. Removal of these participants did not affect any of the significant and non-significant results reported. There were no significant differences between the small (N = 32) and large (N = 36) portion size exposure conditions for age, BMI, dietary restraint, disinhibition, baseline hunger or how often participants reported eating crisps (all *ps* > .05).

There was a significant difference between the two portion size exposure conditions on perceptions of portion size normality [t (66) = 2.5, p = .015, *d* = 0.60]. After exposure to larger portions of crisps, participants believed a normal serving of crisps would be larger than did participants exposed to the image of smaller portion of crisps. See Table 3. There was, however, no significant effect of exposure condition on grams of crisps consumed [t (66) = 1.1, p = .27, *d* = .27]. Participants in the large portion size exposure condition (M = 13.8 g) consumed a similar amount of crisps as participants in the small portion size exposure condition (M = 11.6 g). See Table 3.

### 4.6 Conclusions

In line with studies 1 and 2, exposure to larger (as opposed to smaller) portion sizes of crisps resulted in participants believing that a normal sized portion of crisps was larger. However, we did not find any evidence that this exposure effect significantly influenced the amount of crisps that participants then freely selected and consumed. We did note that crisp consumption was relatively low across all participants (approximately half the amount of a standard bag of the crisps used). Although we assumed that crisps are a fairly common snack food to consume during most times of the day, the relatively low food intake observed in study 3 could have been a result of testing participants during the morning. An additional limitation of study 3 was that we recruited predominantly healthy weight females, so it is not clear whether a similar pattern of results would be observed for males or females of different weight statuses.

## 5 General discussion

Across three studies, we examined the effect of visually exposing participants to images of large or small portion sizes of food. Exposure to large as opposed to small portion sizes of a food resulted in participants perceiving a normal portion of that food to be larger (relative to an intermediate sized portion). In studies 1 and 2 exposure to larger portion sizes of a food also resulted in participants reporting a larger ideal portion size of that food. However, in study 3 we did not find evidence that exposure to larger portion sizes resulted in participants self-serving and then consuming more of a snack food.

The present research indicates that mere visual exposure to larger portions of a food may serve to recalibrate perceptions of what is a ‘normal’ serving of that food. Our interpretation of this effect is that when individuals make judgements about the normality of a stimulus (the size of a food portion), evaluations are skewed by the examples (in this case sizes) of that stimulus that an individual has recently encountered (as in the present studies) and/or frequently encounters (see Oldham & Robinson, 2015). An interpretation which is in line with this is a recent anchoring and adjustment interpretation of portion size effects suggested by Marchiori et al. (2014). Thus, exposure or ‘anchoring’ to larger portion sizes may result in larger becomes synonymous with ‘normal’ (Oldham and Robinson, 2015, Robinson and Kirkham, 2014). If this interpretation is correct, then our findings may also explain why individuals sometimes have inaccurate perceptions of what constitutes a standard serving of food and often believe a standard serving of food is larger than the recommended serving amount (Bryant and Dundes, 2005, Schwartz and Byrd-Bredbenner, 2006); because portion sizes have increased in recent times, visual perceptions may have become recalibrated. Of course, the present studies only examined perceptions of portion size normality immediately after visual exposure, so it is unclear how long such effects last for and whether they translate out of the laboratory. Thus, these interpretations are speculative at present.

Although exposure to larger portion sizes influenced self-reported ideal meal size (studies 1 and 2), in study 3 we did not find that exposure to images of larger portions of crisps influenced actual consumption (of crisps). We had hypothesised that altering perceptions of portion size normality may influence food consumption because a number of studies indicate that individuals often eat in line with what they believe to be the ‘norm’ or a ‘normal’ amount to consume (Kerameas et al., 2014, Robinson et al., 2014). Therefore, the effects we observed on self-reported ideal portion size but not on actual meal size warrant further attention. One possible explanation is that when individuals make hypothetical decisions about portion size (‘if I were to eat this as a meal in the future’), perceptions of portion size normality are weighted more strongly than when participants are asked to make an actual portion size selection and consume a meal. When actually selecting and consuming a meal in the present moment, a number of other factors may be more likely to be weighted in decision making, such as the time since last eating (Sadoul, Schuring, Mela, & Peters, 2014) or satiety requirements (Brunstrom & Shakeshaft, 2009), which may result in a smaller influence of portion size normality. A further explanation is based the types of food used; in studies 1 and 2 we used an amorphous main meal food type (spaghetti bolognese) whilst in study 3 we used a non-amorphous snack food (crisps). Snack food intake is thought to be influenced by perceptions of portion size normality (Robinson et al., 2013, Vartanian et al., 2013). However, it is conceivable that because the food used in study 3 were in discrete units (individual crisps), participants may have had pre-existing beliefs about what is an appropriate number of units to eat and this may have reduced the influence that visual exposure to larger portions had on behaviour. Likewise, it may be easier to count units of food and this itself may impact on evaluations of portion size normality. A final explanation is that the shift in portion size normality we observed in studies 1 and 2 (average *d* = 0.92) was larger than the shift observed in study 3 (average *d* = 0.60) which may have been conducive to a change in self-reported ideal meal size (studies 1 and 2) but not actual meal size (study 3).

### 5.1 Limitations and future directions

A limitation of the present studies was that when selecting the images of smaller vs. larger portion sizes we did not attempt to precisely match volume or energy content of food across studies (e.g. the relative size difference between small and large portions differed in study 3 compared to study 1 and 2). Moreover, the ‘intermediate’ portion size that participants made evaluations about was not exactly half way between the size of the large and small portions (in terms of volume of food or energy content) as we chose these images based on what appeared intermediate by eye. Greater consistency across studies would have aided interpretation of findings. We did however find that across all three studies visual exposure to larger portions did affect evaluations made about our selected ‘intermediate’ portions. Careful consideration about what constitutes (or appears to constitute) a ‘large’, ‘small’ or ‘intermediate’ sized portion of food will be important for future work.

Notwithstanding the above, the ‘real world’ relevance of our findings warrants attention. Here we briefly exposed participants to images of large or small portions and measured portion size normality immediately afterwards. We therefore do not know how long lasting these effects may be. It may be the case that brief visual exposure only temporarily alters perceptions of what constitutes a normal sized portion and more frequent exposure is required to produce meaningful long term changes to perceptions of portion size. For example, one potential interpretation of our results is that if portion sizes were decreased for a food type, then over time (and as a result of repeated exposure) this may cause consumers to recalibrate their visual perceptions of what a normal portion of that food looks like. Thus, understanding the long term consequences of being exposed to different food portion sizes would now be valuable. Likewise, in the present studies we did not thoroughly examine whether visual exposure effects transfer (e.g. whether exposure to large portions of a variety of food types influences perceptions of portion size normality for a distinct food). Therefore, further work specifically examining potential ‘transfer’ effects might be helpful. Examining whether there are conditions under which altering visual perceptions of portion size normality influences food consumption would also be valuable. In the present studies we did not find evidence in support of this, but as discussed this may have been a consequence of the food types used, exposure duration and/or the magnitude of change in portion size normality we observed.

### 5.2 Conclusions

Visual exposure to larger portion sizes may adjust visual perceptions of what constitutes a ‘normal’ sized portion. However, we did not find evidence that visual exposure to larger portions altered snack food intake.

## Figures and Tables

**Table 1:** Perceived normality and ideal portion size scores for food in study 1.

	Small portion condition (N = 52)	Control condition (N = 51)	Large portion condition (N = 47)
Perceived normality of spaghetti bolognese	3.8 (1.3)^a^	4.7 (1.3)^a^	5.3 (1.6)^a^
Ideal portion size for spaghetti bolognese	3.8 (1.6)^b^	4.7 (1.6)^b^	5.5 (1.4)^b^
Perceived normality of chicken curry and rice	5.0 (1.1)	5.2 (1.7)	5.5 (1.7)
Ideal portion size for chicken curry and rice	4.6 (1.8)	4.6 (1.8)	4.9 (2.1)

Values are means (standard deviations) on a 0–10 visual analogue scale. Perceived normality question: ‘a normal serving of …. would be’, anchors: 0 (a lot smaller) and 10 (a lot bigger). Ideal portion size question: ‘If I were to eat this for an evening meal, I would want a portion size that was’, anchors: 0 (a lot smaller) and 10 (a lot bigger).

^a,b^ Same superscript denotes significant between condition difference (p < .05).

**Table 2:** Perceived normality and ideal portion size scores for food in study 2.

	Small portion condition (N = 52)	Large portion condition (N = 47)
Perceived normality of spaghetti bolognese	4.5 (1.3)^a^	5.6 (1.5)^a^
Ideal portion size for spaghetti bolognese	4.3 (1.6)^b^	5.9 (1.4)^b^
Perceived normality of chicken curry and rice	5.0 (1.3)^c^	6.0 (1.4)^c^
Ideal portion size for chicken curry and rice	5.1 (2.1)	5.9 (1.7)

Values are means (standard deviations) on a 0–10 visual analogue scale. Perceived normality question: ‘a normal serving of …. would be’, anchors: 0 (a lot smaller) and 10 (a lot bigger). Ideal portion size question: ‘If I were to eat this for an evening meal, I would want a portion size that was’, anchors: 0 (a lot smaller) and 10 (a lot bigger).

^a,b,c^ Same superscript denotes significant between condition difference (p < .05).

**Table 3:** Perceived normality of intermediate portion size of crisps and crisp intake in study 3.

	Small portion condition (N = 32)	Large portion condition (N = 36)
Perceived normality of intermediate portion size^a^	5.3 (1.6)^b^	6.1 (1.1)^b^
Grams of crisps consumed	11.6 (6.7)	13.8 (9.2)

a For the intermediate sized portion of crisps, participants rated ‘a normal serving of crisps would be...’ ‘a lot smaller than this’ and ‘a lot bigger than this’ (end-point anchors) on a 0–10 cm VAS.

b Indicates significant between condition difference (p < .05).
